# Prediction of jumbo drill penetration rate in underground mines using various machine learning approaches and traditional models

**DOI:** 10.1038/s41598-024-59753-6

**Published:** 2024-04-18

**Authors:** Sasan Heydari, Seyed Hadi Hoseinie, Raheb Bagherpour

**Affiliations:** Department of Mining Engineering, Isfahan University of Technology, Isfahan, 84156-83111 Iran

**Keywords:** Penetration rate prediction, The Rock Mass Drillability Index (RDi), Traditional models, Multilayer perceptron neural networks (MLP), Support Vector Regression (SVR), Random Forests (RF), Environmental social sciences, Engineering

## Abstract

Estimating penetration rates of Jumbo drills is crucial for optimizing underground mining drilling processes, aiming to reduce costs and time. This study investigates various regression and machine learning methods, including Multilayer Perceptron (MLP), Support Vector Regression (SVR), and Random Forests (RF), to predict the penetration rates (ROP) using multivariate inputs such as operation parameters and rock mass characteristics. The Rock Mass Drillability Index (RDi), incorporating both intact rock properties and structural parameters, was utilized to characterize the rock mass. The dataset was split into 80% for training and 20% for testing. Performance metrics including correlation coefficient (R^2^), variance accounted for (VAF), mean absolute error (MAE), mean absolute percentage error (MAPE), and root mean square error (RMSE) were calculated for each method to evaluate the accuracy of the predictions. SVR exhibited the best prediction performance for ROP, achieving the highest R2, lowest RMSE, MAE, and MAPE, as well as the largest VAF values of 0.94, 0.15, 0.11, 4.84, and 94.13 during training, and 0.91, 0.19, 0.13, 6.02, and 91.11 during testing, respectively. With this high accuracy, we conclude that the proposed machine learning algorithms are valuable and efficient predictors for estimating jumbo drill penetration rates in underground mining operations.

## Introduction

**Figure 1:**
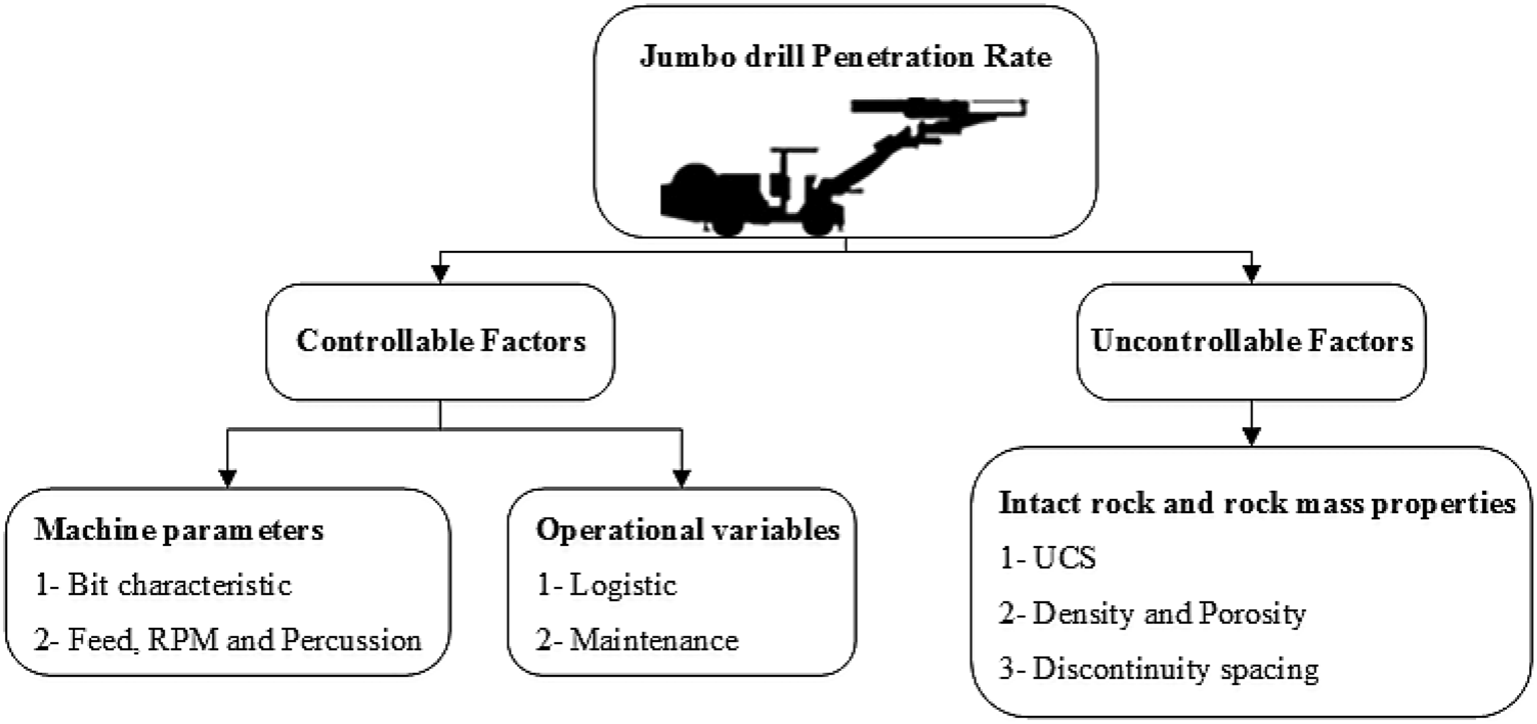
The most important factors influencing drilling performance.

The drill and blast method is the most important method used in underground excavation. In the drill and blast method in tunneling, drilling constitutes the largest cost and time^1^. In drilling operations, many factors such as geological, geotechnical, operational, and machine characteristics, affect drilling performance. These parameters can be generally classified into two major groups controllable and uncontrollable parameters^2,3^. Operational factors and machine characteristics are variables that can be controlled but geological and geotechnical conditions are unique to each site and cannot be easily altered^4^. The key factors that impact the Rate of Penetration (ROP) are shown in Fig. 1.

Predicting the ROP based on drilling variables is essential aiming to maximize ROP or minimize total time or cost. For that, the accuracy of the ROP model is crucial^4^. Despite the importance of predicting ROP for better drilling efficiency, accurately establishing a prediction model is challenging^5^. The empirical approach, commonly used to study penetration rates, utilizes field data and is developed for varying ground conditions. Various studies conducted by researchers on percussive and rotary drilling have revealed that the rate of penetration is contingent upon the properties of the rock^6^. The relationship between rock properties and ROP is complex and nonlinear. Many researchers developed statistical models to predict ROP based on the experimental data^7–17^. Various statistical models and classification systems have been developed to predict penetration rate, but they often lack generalizability across different drilling conditions^18^. Existing methods may not accurately predict ROP in different geological settings or with different drilling rigs^19–24^. Many of these statistical relations only show the effect of different rock parameters on ROP separately. Few of them can predict ROP based on few rock properties.The Norwegian Institute of Technology introduced the Drilling Rate Index (DRI) for assessing drillability in percussive drilling^25^. Linear predictive equations were developed correlating rate of penetration with various rock properties such as compressive strength, tensile strength, and Young's modulus. Despite investigating numerous rock mechanical properties, researchers found that only compressive strength, tensile strength, and Young's modulus were indicative parameters for predicting penetration rate^26^. Furthermore, researchers noted that penetration rate closely relates to bulk density, compressive strength, apparent porosity, P-wave velocity, and Schmidt hammer value^27^. Correlations between ROP and different rock hardness test results were demonstrated, with compressive strength and tensile strength showing a strong correlation with ROP^9,16^. Multiple regression analysis was employed to construct PR models for various drill types, including rotary, Down-the-Hole (DTH), and hydraulic top hammer drills^17^. Moreover, a new index named the Rock Penetrability Index (RPi) was developed, considering factors like UCS, Schimazek’s F-abrasivity, Mohs hardness, rock texture, grain size, and Young’s modulus^28,29^. Density was also identified as an important parameter for predicting ROP^12^. Apart from intrinsic rock properties, discontinuities, such as the Rock Quality Designation (RQD), significantly affect the cutting performance^30^. Additionally, researchers have presented a drillability prediction model based on parameters such as UCS, Schmidt hammer hardness value, quartz content, fragment size, alternation, joint dip, bit rotational speed, and thrust^31^. Furthermore, studies have explored the relationship between ROP and drill rig operational parameters, with theoretical and experimental investigations suggesting that ROP can be predicted by specific energy and certain design and operational parameters of the drilling machine^32^. Relationships between ROP and drilling thrust, as well as the influence of rock strength, drill rig power, drill bit shape, and geological discontinuities on ROP, have also been highlighted^33^. Some of the research findings include empirical models based on the uniaxial compressive strength of rocks, the impact of geological features on penetration rate, and the use of Artificial intelligence techniques to predict rock classification around tunnels. The studies also cover topics related to percussive and rotary drilling, the influence of different parameters on penetration rate, and the development of new models for predicting drilling performance^34–51^.

Statistical approaches in ROP prediction suggest relationships between input and output parameters, but these methods may not always effectively handle non-linear and complex problems^52,53^. The traditional multiple regression model lacks adequate prediction stability and struggles to solve nonlinear problems due to the impact of multicollinearity among independent variables^54^. Artificial Intelligence Based models have been introduced to address these limitations and, over time, have demonstrated improvements in prediction accuracy^55^.

The advancement of information technology has led to the emergence of intelligent drilling and completion technology in the field. Currently, there is a growing trend in the application of various machine learning techniques for estimating ROP as they continue to evolve and advance rapidly^56^. Due to the need for frequent recalibration with traditional physics-based models based on auxiliary data, machine learning models strive to overcome these challenges by utilizing data to identify correlations among various drilling variables^57^. Machine learning algorithms can take any number of measured variables as inputs, making them a powerful tool for ROP modeling^4^. ML algorithms have an advantage over analytical ROP models as they offer flexibility in model form, which allows them to effectively segment the drilling operational parameter space. Unlike analytical models, ML algorithms do not require predefined equations, as hyperparameters specific to each algorithm can control the model architecture. However, increased model complexity can lead to reduced interpretability and an increased risk of overfitting. Also, in the presence of multiple collinearity between input variables, Methods like neural networks often encounter challenges such as an unstable learning process and slow convergence speed in calculations^58^.

The application of machine learning techniques in underground mines extends beyond the study of ROP (Rate of Penetration), and nowadays, it is also utilized in other fields of engineering. With the widespread use of jumbodrills equipped with Measurement While Drilling (MWD) systems, it has become customary to employ machine learning and deep learning techniques for classifying the rock mass in tunnels, Predictive modeling of drilling rate, as well as detecting deviations in bore holes within the face^51^. In addition to the aforementioned applications, given the paramount importance of safety in underground mines, the utilization of these techniques in various areas such as fire prediction, column stability, androckburst in underground engineering structures is rapidly expanding^59–61^.

One of the early applications of Machine Learning for predicting drilling parameters, was presented by Arehart 1990^62^. Arehart employed Artificial neural networks (ANN) to predict a crucial drill bit parameter, specifically bit wear. Subsequently, Bilgesu et al. published the first study applying ANN for predicting the ROP^63^. In drilling, machine learning techniques such as ANN, random forests, and other regression and classification methods, along with deep learning methods, have demonstrated numerous applications applications. They have proven to be advantageous^64^. The findings suggest that artificial neural networks are the most commonly used machine learning technique for managing the rate of penetration. Among ANNs, basic models outperform modified versions in this context. However, while modified ANNs demonstrate greater accuracy in predicting ROP, they are not superior to other machine learning methods such as linear regression (LR) and random forest (RF) in making highly accurate predictions. These alternative approaches have been shown to be effective and practical in compensating for the limitations of ANNs in ROP management^3,65^. Moreover, similar studies have been conducted to predict drilling rate using machine learning techniques such as random forest. However, these models encounter constraints due to the utilization of laboratory samples. It is imperative that these models undergo testing in the field with real-time drilling data to gain further insight into their performance in practical scenarios^66,67^.

After reviewing the literature on the subject, it was discovered that only a few researchers have developed relationships to predict the penetration rate of jumbodrills. Kahraman calculated the performance prediction of a jumbo drill based on intact rock and rock mass properties^45^. The relationship he presented focuses on the discontinuities within the rock mass. Shen found relationships between the drilling rate and drilling parameters based on drilling data. To explore the variations in drilling parameters, drilling tests were conducted on a rock block. His relations do not take into account the characteristics of the rock mass^68^. Su developed multiple regression models using UCS, DRI, impact energy, blows per minute of the piston, hole area, and some rock properties for predicting the penetration rate and specific energy of drilling^69^.

Prasad developed an experimental methodology to measure the expected bit life along with ROP using a single test method and Compendious Index for Drillability^70^.

As far as the available literature suggests, there have been no attempts made to conduct a comprehensive review solely focusing on the recent advancements of machine learning techniques for predicting the jumbo drill penetration rate. Thus, the main aim of this paper is to fill this research gap and address crucial queries related to intelligent prediction techniques for jumbo drill's ROP. In this paper, in addition to regression methods, three machine learning methods, Multilayer perceptron (MLP), Support Vector Regression (SVR), and Random Forests (RF), are used to estimate ROP. All calculations in this paper were performed using a custom program code written in the R Statistical Programming Language.

## Case study and data collection

The objective of this paper is to develop an empirical model with machine learning algorithms that can predict the penetration rate of jumbo drills in underground mines. The collection of real field data constitutes a significant and essential part of this study. The models were developed using real data collected from seven underground lead and zinc mines that were extracted by sublevel stoping mining method located in the Irankouh and Sormeh mining districts of Iran. To create the models, the study considered six parameters, which relate to drilling and rock properties. In these mines, the tunnel face area is about 16–24 square meters and contains approximately 30 ~ 35 blasting holes per face. The jumbo drills used for the study included four different brands, with drill bit diameters of 51 and 64 mm. The collected parameters are described in Table 1.

**Table 1:** Description of parameter symbols and values used in the model.

Parameter	Unit	Symbol	Description
Feed pressure	bar	P_F_	The hydraulic pressure inside the cylinders required to keep the bit in contact with the bottom of the hole
Rotation pressure	bar	P_R_	The pressure of the bit against the rock to maintain the required rotation
Hammer pressure	bar	P_P_	The measurement of the impact pressure of the bit against the rock mass
Borehole Diameter	mm	D_h_	Borehole Diameter, The diameter of the bits used to drill the boreholes is 51 and 64 mm
Rock mass drillability index	–	RDi	Rock mass drillability index, include physical parameters of the rock materia, strength parameters of the rock material and structural parameters of the rock mass
The rate of penetration	m/min	ROP	The result of dividing the drilling time by the length of the borehole

### Relationship between rock mass parameters and ROP

Having reviewed the studies conducted thus far, it becomes apparent that several rock mass parameters significantly influence drilling operations. These include the origin of the rock formation, Mohs hardness, texture (grain shape and size), porosity, density, abrasiveness, rigidity, P-wave velocity, elasticity, plasticity, UCS (point load index and Schmidt hammer), tensile strength, and structural parameters (such as joints, cracks, and bedding) alongside RQD.

Rock classification poses a challenge due to the selection of parameters deemed most significant. No single parameter or index can comprehensively describe a jointed rock mass for engineering purposes. Consequently, it is impractical to incorporate all parameters into a classification system. In the rock mass drillability index classification scheme (RDi), four guiding principles have been adopted: a limited number of parameters, avoidance of equivalent parameters, categorization of parameters into groups, and field applicability of the classification. Adhering to these principles, the RDi assessment focuses on three categories of parameters: physical parameters of the rock material: texture and grain size, (b) strength parameters of the rock material: UCS and Mohs hardness, and (c) structural parameters of the rock mass: joint spacing, joint filling and aperture, and joint dipping. Except for UCS, all parameters are readily measurable in the field. Since it is not possible to measure the UCS in the field, one can use point load index and Schmidt hammer results as equivalent values for the UCS^27^.

Based on the points mentioned, there are many uncontrollable parameters that affect the penetration rate. However, incorporating all these variables is complex, time-consuming, and costly. Therefore, this paper employs the Rock Mass Drilling Index (RDi) as a representative of rock mass properties. RDi encompasses both intact rock properties and structural parameters, serving as a comprehensive indicator of rock mass characteristics.

To date, there has been no index used for underground mining that comprehensively encompasses all rock mass characteristics. The RDi, developed by Hosseini, provides a qualitative representation of the drilling rate by considering both intact rock properties and structural parameters of the rock mass^27^. This classification system is presented in Table 2, and the qualitative predictions are shown in Table 3. By summing the ratings in Table 2, a qualitative prediction of the rock mass drilling rate can be calculated.

**Table 2:** Rock mass drillability index (RDi) classification^27^.

Texture	Porous	Fragmental	Granitoid	Porphyritic	Dense
Grain size	–	> 5 mm	2–5 mm	0.05–1 & 2–5 mm	0.05–1 mm
Rating	15	10	7	4	1
Mohs Hardness	1–3	3–4.5	4.5–6	6–7	> 7
Description	Very soft–soft	Comparatively soft	Comparatively hard	Hard	Very hard
Rating	18	13	9	4	1
UCS (MPa)	1–25	25–50	50–100	100–200	> 200
Description	Very low strength	Low strength	Average strength	High strength	Very high strength
Rating	22	16	11	6	2
Joints Spacing	> 2 m	1–2 m	0.5–1 m	0.15–0.5 m	0–0.15 m
Rating	18	13	9	5	1
Joint aperture & filling	Closed joint 0–2 mm	> 20 mm	12–20 mm	9–12 mm	2–9 mm
Rating	15	10	7	4	1
The angle between the joint & borehole axis	70°–90°	55°–70°	35°–55°	20°–35°	0°–20°
Rating	12	8	6	3	1

**Table 3:** Qualitative prediction of the penetration rate of drilling in the rock mass using RDi^27^.

RDi	7–20	20–40	40–60	60–80	80–100
Prediction of drilling rate	Slow	Slow–medium	Medium	Medium–fast	Fast

**Figure 2:**
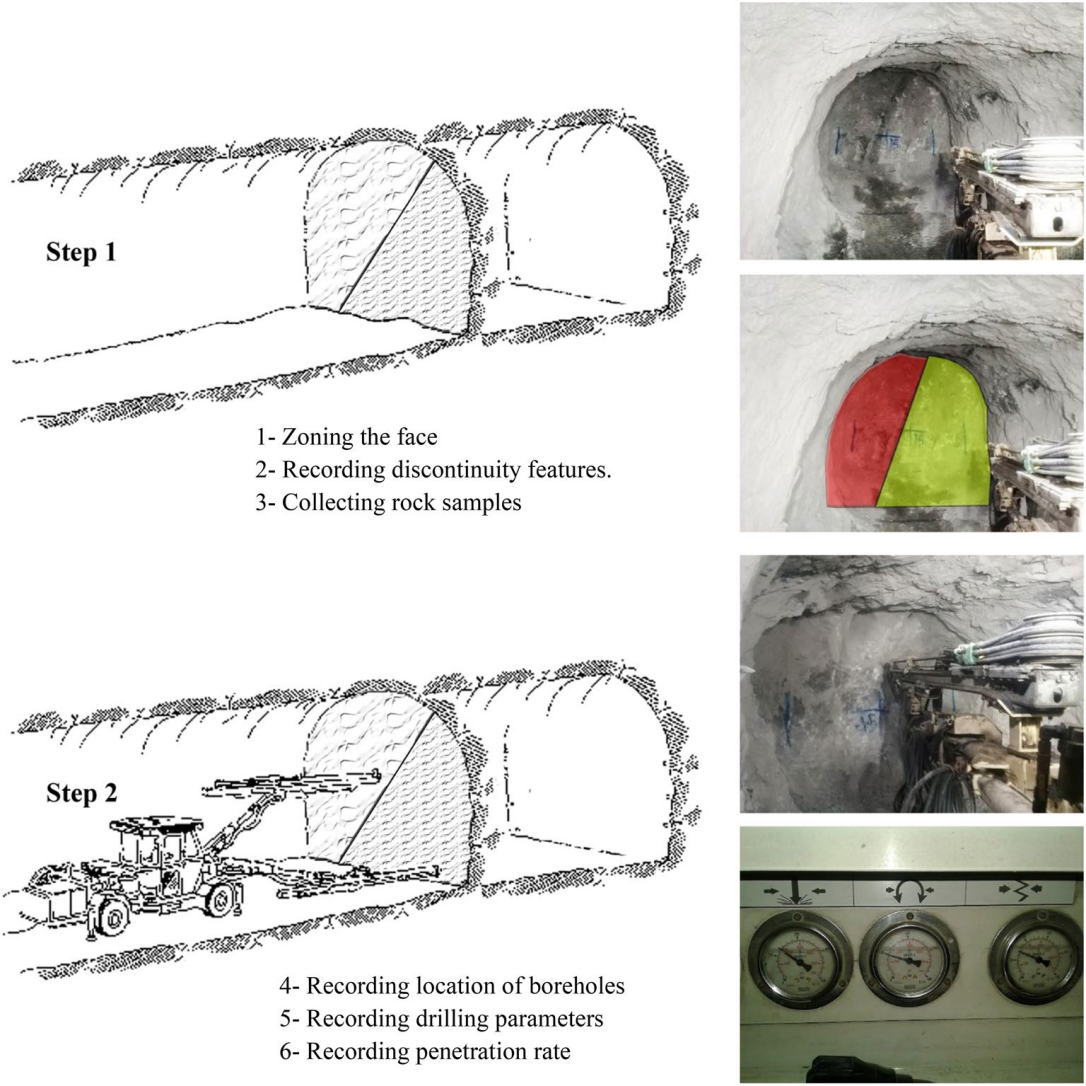
Different steps of data collection in this paper.

Before commencing tunnel drilling, relevant information such as depth, cross-sectional area, water presence, and other parameters was gathered. Prior to jumbo drill operations in each tunnel face, data on discontinuity properties were collected, comprising joint spacing, joint aperture and filling, and the angle between the joint and borehole axis. A select number of suitable samples from each face underwent laboratory studies to determine the physical and mechanical properties of the rock. A total of 737 boreholes in 26 faces, equivalent to more than 2400 m, were considered for this study. Uniaxial compressive strength was calculated following International Society for Rock Mechanics (ISRM) guidelines (RTH 325–89), and density and porosity tests were conducted in accordance with ISRM Suggested Methods. Additional assessments included grain size, texture, and Mohs hardness determination. Each face was divided into distinct zones based on the rock mass drillability index (Refer to Fig. 2), and these zones were designated for borehole drilling. Data related to drilling in each specific area were recorded.

**Figure 3:**
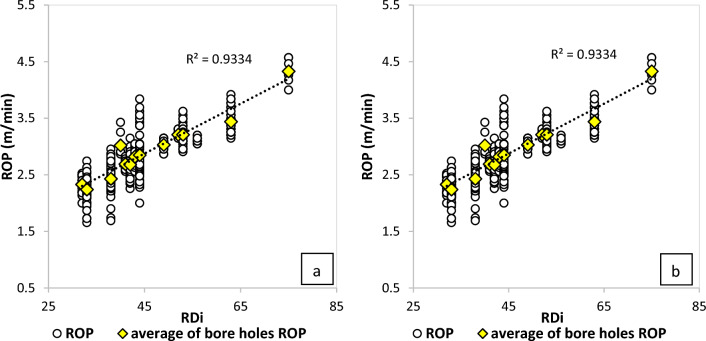
Relationship between RDi and all available datasets penetration rate and the average of many numbers of blasting holes (**a**) 51 mm (**b**) 64 mm.

The Rock Mass Drillability Index (RDi) parameters collected from tunnels were rated as per Table 2 to classify rock mass drillability (see Table 3). To evaluate the correlation between RDi and the Rate of Penetration (ROP), correlation diagrams were created for two borehole diameters (51 and 64 mm) with respect to penetration rate (Fig. 3). The analysis revealed a linear increase in penetration rate with higher RDi values in the rock mass. This linear relationship was chosen based on the highest correlation coefficients among linear, logarithmic, power, and exponential functions. The variability in penetration rate for each RDi value is attributed to differences in machine operating parameters. To clarify the correlation between these two factors, the data related to each RDi class was clustered. Each solid point in Fig. 3 represents the average of multiple blasting holes, ranging from 32 to 75, with one point representing each group.

## Data preprocessing

To ensure accurate model training, it is crucial to preprocess raw data due to its high level of noise and outlier data. Neglecting to remove outliers and reduce noise can impede the model learning process and prolong training time. At this stage, the collected data undergoes review and analysis to prepare for program entry, with 80% of the data used for training and 20% for testing randomly. To ensure accurate data analysis, it is critical to take two necessary actions: First, data analysis, verification of their accuracy and precision and Second, data matching; to avoid scattering and place all the data in a specific interval. Non-quantitative data can be handled using various techniques. Improper scaling can cause regression analysis to misestimate the significance of each variable, and deleting or averaging out-of-range data can be used as a solution. This study employs the IQR method, one of the most commonly used outlier labeling techniques. To handle extreme values that can behave as outliers in a noisy dataset, it is necessary to perform outlier labeling before processing. The IQR method is considered one of the best approaches, which takes the interquartile range (IQR) into account for labeling outliers, using Eq. (1):

1 $$ {\text{IQR}} = {\text{Q}}_{3} - {\text{Q}}_{1} $$

**Figure 4:**
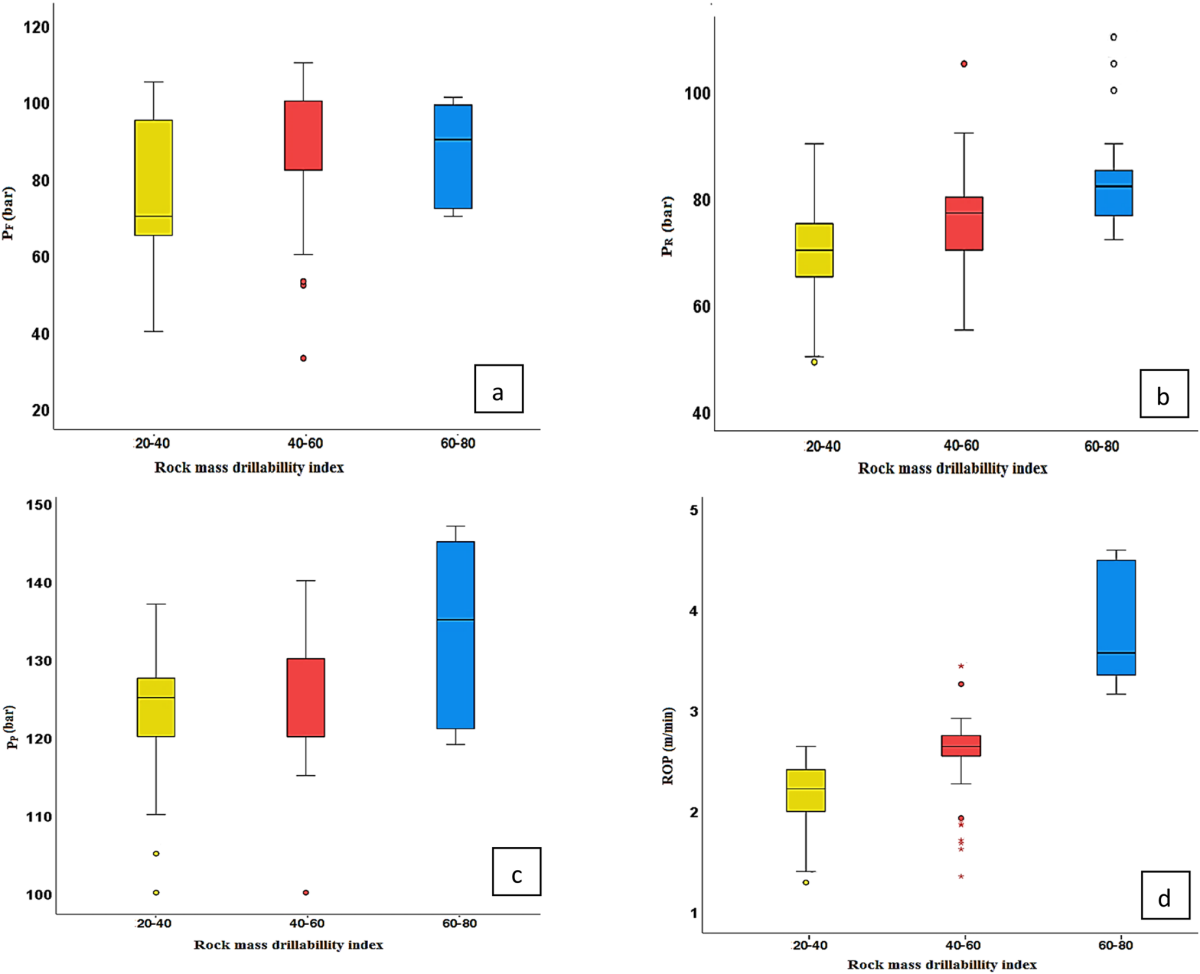
Drilling parameter box diagrams for each RDi. (**a**) Feed pressure (P_F_, bar); (**b**) Rotation pressure (P_R_, bar); (**c**) Hammer pressure (P_P_, bar); (**d**) The rate of penetration (ROP, m/min).

In Eq. (1), Q_3_ represents the 3rd quartile, and Q_1_ represents the 1st quartile of the data. To determine the upper and lower limits of the extreme boundaries, the IQR value is multiplied by a factor and then subtracted from Q_1_ and added to Q_3_. The most commonly used factor for this is 1.5. To analyze the sample data outliers, box diagrams were drawn according to the drilling parameter and rock mass drillability index classification that is shown in Fig. 4. In Fig. 4, under different Rock mass Drillability index, all four drilling parameters contained outliers. To eliminate the effects of the outliers, the average value of the parameter was used to replace the outlier of data in this paper. The changes in data characteristics before and after cleaning are compared in Table 4, after cleaning.

**Table 4:** Comparison of the data characteristics before and after cleaning.

RDi	Index	P_F_		P_R_		P_P_		ROP	
		Before	After	Before	After	Before	After	Before	After
20–40	mean	80.83	80.90	69.96	69.75	125.51	125.61	2.07	2.08
	std	16.02	15.66	10.00	9.49	7.09	6.81	0.40	0.40
	min	40	50	49	50	100	105	1.28	1.28
	max	105	105	105	90	140	140	2.95	2.95
40–60	mean	85.05	86.92	78.36	78.61	124.76	125.30	2.74	2.78
	std	16.99	14.29	12.93	11.22	7.42	6.35	0.45	0.37
	min	33	38	55	55	100	110	1.34	1.85
	max	110	110	115	102	145	145	3.84	3.69
60–80	mean	87.15	88.88	82.62	80.15	133.78	133.45	3.78	3.81
	std	11.69	10.88	9.15	4.42	11.01	10.73	0.52	0.51
	min	70	70	72	72	119	119	3.15	3.15
	max	101	101	110	90	147	147	4.58	4.58

After data cleaning; to avoid scattering and place all the data in a specific interval, in the field of machine learning, different evaluation indexes (that is, different features in feature vectors are described as different evaluation indexes) often have different dimensional and dimensional units, which will affect the results of data analysis. In order to eliminate the dimensional influence between indexes, data normalization is required.

Normalization is a technique used to limit input data to a specific range, often between 0 and 1. In this study, the data were normalized using the min–max normalization method, which is defined in Eq. (2):

2 $$ x^{\prime} = \left( {{\text{x}} - {\text{x}}_{\min } } \right)/\left( {{\text{x}}_{\max } - {\text{x}}_{\min } } \right) $$

In Eq. (2), x represents the value of the original data, x_max_ represents the maximum value of the original data, x_min_ represents the minimum value of the original data, and x' represents the normalized value.

## Validation of the models

In computational mechanics, several metrics are available in statistics. This research implements five performance metrics. The testing performance is assessed based on five statistical performance criteria, namely determination coefficient (R^2^), variance accounted for (VAF), mean absolute error (MAE), mean absolute percentage error (MAPE), and root mean square error (RMSE). VAF can be used to evaluate the variance proportion of the variables, while RMSE, MAE, and MAPE are frequently used to compare the prediction errors of different models—the lower the RMSE, MAE, and MAPE, the better the model performs. These statistical indices are expressed as follows (Eqs. 3–6)^71–73^:

3 $${\text{RMSE}}= \sqrt{\frac{\sum_{{\text{i}}}^{{\text{n}}}{({{\text{T}}}_{{\text{i}}}-{{\text{O}}}_{{\text{i}}})}^{2}}{{\text{n}}}}$$

4 $${\text{MAE}}= \frac{\sum_{{\text{i}}=1}^{{\text{n}}}\left|{{\text{T}}}_{{\text{i}}}-{{\text{O}}}_{{\text{i}}}\right|}{{\text{n}}}$$

5 $${\text{MAPE}}= \frac{100}{{\text{n}}}\times \sum_{{\text{i}}=1}^{{\text{n}}}\left|\frac{{{\text{T}}}_{{\text{i}}}- {{\text{O}}}_{{\text{i}}}}{{{\text{T}}}_{{\text{i}}}}\right|$$

6 $${\text{VAF}}= \left[1-\frac{\mathrm{var }({{\text{T}}}_{{\text{i}}}-{{\text{O}}}_{{\text{i}}})}{\mathrm{var }({{\text{T}}}_{{\text{i}}})}\right]\times 100$$

where var denotes the variance, ${{\text{T}}}_{{\text{i}}}$ and ${{\text{O}}}_{{\text{i}}}$ are the measured and predicted values, n is the sample size.

## A penetration rate model for Jumbo drills

### Drilling performance assessment using non-linear regression analyses

Assessment of geo-mechanical properties of rock formations plays a crucial role in determining the efficacy of rock drilling, as well as predicting drilling-related costs, timing, and productivity. Predictive modeling techniques, such as regression analysis, can provide valuable insights into the relationships between independent and dependent variables.

In order to ensure that a model is capable of making accurate predictions on unseen data, it is necessary for it to perform well not only on the data that it was trained on but also on novel data. To accomplish this, the dataset is partitioned randomly into two subsets, with 80% of the data used for training and the remaining 20% used for testing.

**Figure 5:**
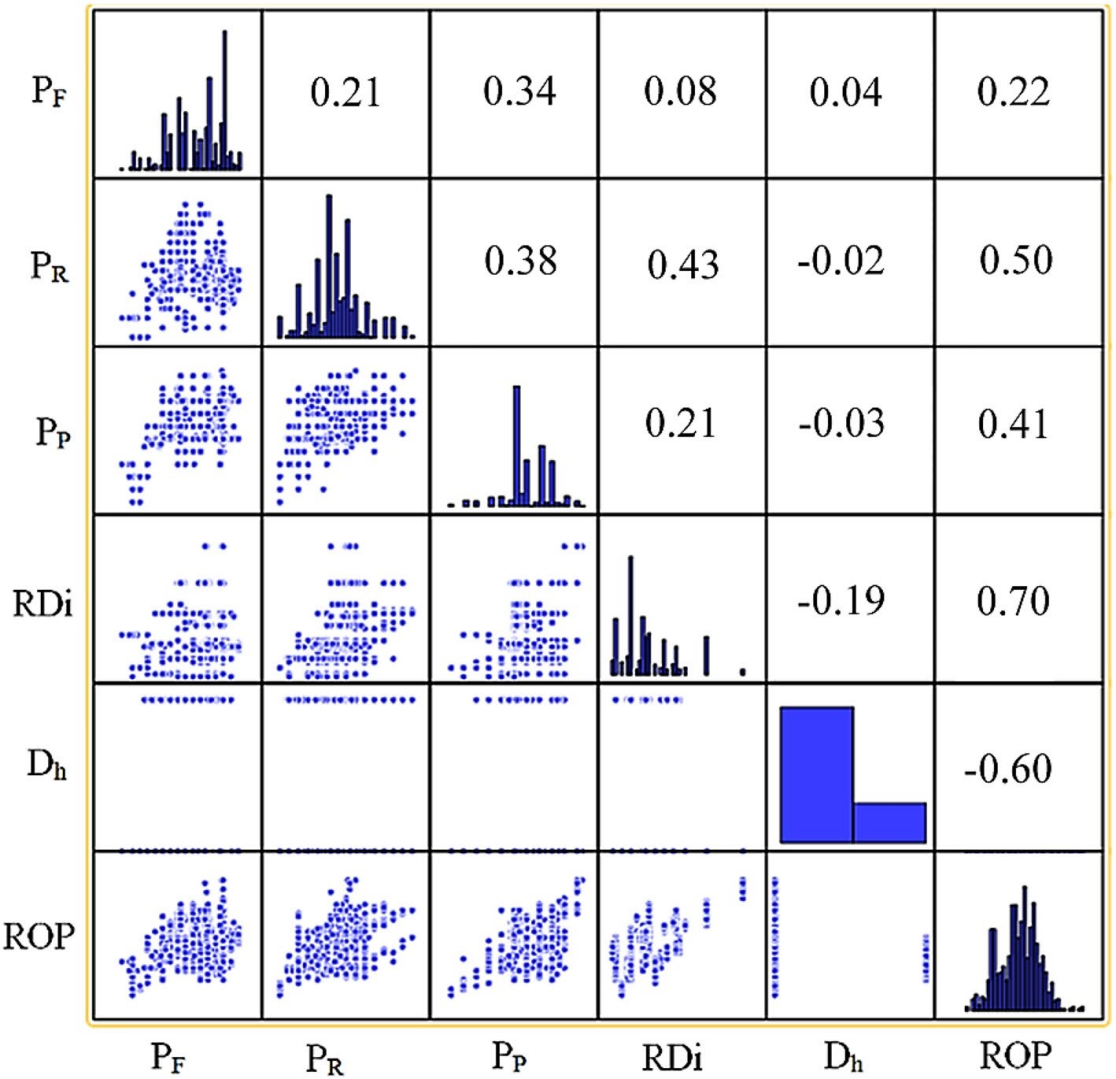
Correlogram of the dataset.

Initially, we conducted several basic regression analyses utilizing linear, logarithmic, power, and exponential functions to establish the correlation between each independent variable and the rate of penetration. The coefficients of determination resulting from these basic regression analyses are compiled in Table 5. Based on the findings of the correlation analysis between each independent variable and ROP, it became evident that the rate of penetration cannot be anticipated by a single variable alone. Instead, it is impacted by numerous factors. Consequently, statistical methods and machine learning methods were used to attain more precise predictions of the rate of penetration. The correlogram of the dataset is represented in Fig. 5. In Fig. 5, scatter plots for each pair of variables are presented on the lower left, correlation coefficients are shown on the upper right, and histograms are placed in the middle. It is apparent from Fig. 5 that there was no substantial overlap between the independent variables, and none of them were removed while constructing the model.

**Table 5:** Correlation analysis of rate of penetration and individual parameters.

Parameters	Linear	Logarithmic	Power	Exponential
P_F_	0.13	0.19	0.25	0.18
P_R_	0.45	0.49	0.53	0.46
P_P_	0.27	0.28	0.34	0.31
RDi	0.72	0.72	0.70	0.68

In contrast to the limited scope of traditional linear regression, non-linear regression is capable of estimating models that encompass complex relationships between independent and dependent variables. In this paper, different algorithms were used to describe nonlinear multivariate regression functions, especially focusing on simple regression functions. The resulting models were then compared to identify the most accurate one based on the model development data. Table 6 presents five distinct non-linear multivariable regression equations that were derived using the model development data. As shown in Table 6, the correlation coefficients for these models ranged from 0.82 to 0.87 when assessed solely on the model development datasets. However, when considering the testing datasets, the correlation coefficients were found to be between 0.81 and 0.86.

**Table 6:** Correlation coefficients for non-linear multivariable regression models.

Model	Regression equation	R^2^ (Training)	R^2^ (Testing)
1	${\text{ROP}}=2.39\times \frac{{{\text{P}}}_{{\text{F}}}^{0.11}\times {{\text{P}}}_{{\text{R}}}^{0.3}\times {{\text{P}}}_{{\text{P}}}^{0.6 }\times {{\text{RDi}}}^{0.59 }}{{{\text{D}}}_{{\text{h}}}^{1.73}}$	0.87	0.86
2	${\text{ROP}}=199 {{\text{P}}}_{{\text{F}}}^{0.001}+295 {{\text{P}}}_{{\text{R}}}^{0.002}+254 {{\text{P}}}_{{\text{P}}}^{0.001}+0.07 {{\text{RDi}}}_{ }^{0.006}-744 {{\text{D}}}_{{\text{h}}}^{0.005}$	0.85	0.82
3	ROP = (0.003 P_F_ + 0.2)^2^ + (0.007 ${{\text{P}}}_{{\text{R}}}$ + 0.2)^2^ + (0.008 ${{\text{P}}}_{{\text{P}}}$ − 0.3)^2^ + (0.02 ${\text{RDi}}$ + 0.006)^2^ + (− 0.07 ${{\text{D}}}_{{\text{h}}}$ + 4.6)^2^	0.82	0.81
4	${\text{ROP}}=2.8 -\frac{19.6}{{{\text{P}}}_{{\text{F}}}}-\frac{49.7}{{{\text{P}}}_{{\text{R}}}}-\frac{192}{{{\text{P}}}_{{\text{P}}}}-\frac{73.8}{{\text{RDi}}}+\frac{216.3}{{{\text{D}}}_{{\text{h}}}}$	0.85	0.84
5	${\text{ROP}}=2.824\times {1.002}^{{{\text{P}}}_{{\text{F}}}}\times {1.004}^{{{\text{P}}}_{{\text{R}}}}\times {1.004}^{{{\text{P}}}_{{\text{P}}}}\times {1.012}^{{\text{RDi}}}\times {0.97}^{{{\text{D}}}_{{\text{h}}}}$	0.83	0.81

By examining the developed nonlinear regression models, it was found that the model specified by the below equation is the most accurate model among the models.

7 $${\text{ROP}}=2.39\times \frac{{{\text{P}}}_{{\text{F}}}^{0.11}\times {{\text{P}}}_{{\text{R}}}^{0.3}\times {{\text{P}}}_{{\text{P}}}^{0.6 }\times {{\text{RDi}}}^{0.59 }}{{{\text{D}}}_{{\text{h}}}^{1.73}}$$

**Figure 6:**
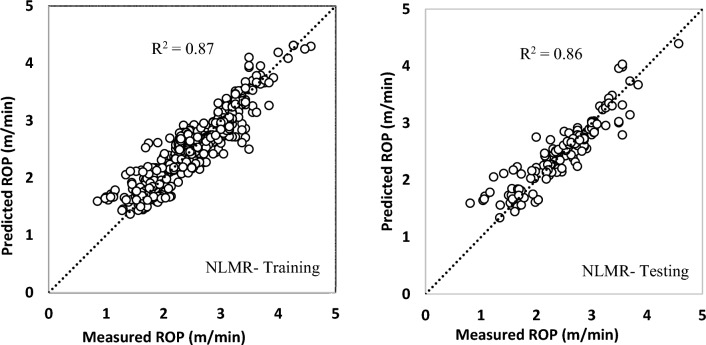
The cross-correlation graph of the nonlinear multiple regression model.

The cross-correlation graph of the nonlinear multiple regression model is presented in Fig. 6.

### Drilling performance assessment using machine learning methods

Model complexity significantly influences the trade-off between bias and variance. Bias arises when complex relationships are oversimplified, and variance measures the sensitivity to dataset variations. In ROP modeling, an analytical model assuming a power law for drilling speed based on a few variables can introduce notable bias due to oversimplification. Analytical ROP models tend to exhibit high bias as they oversimplify the complex drilling process. In contrast, machine learning algorithms allow for the inclusion of numerous input variables. However, increasing model complexity raises the risk of overfitting, which adds variance. Machine learning algorithms provide flexibility in exploring drilling parameters by adapting to the data's characteristics via algorithm-specific hyperparameters rather than predefined equations^4^. Artificial neural networks, support vector machines, and random forests were chosen as the algorithms chosen for this paper based on performance and applicability to a wide range of problems.

**Figure 7:**
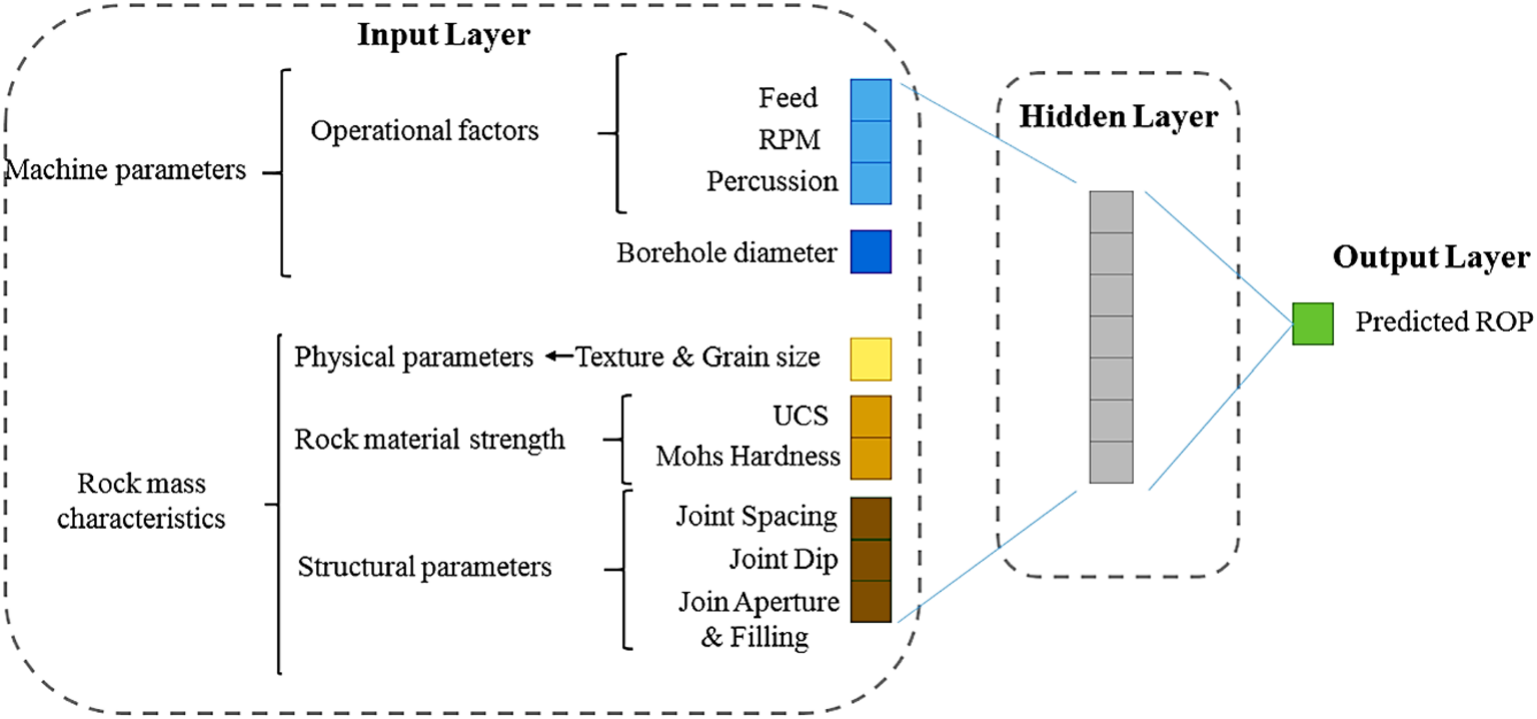
The architecture of the MLP model used in the paper.

**Figure 8:**
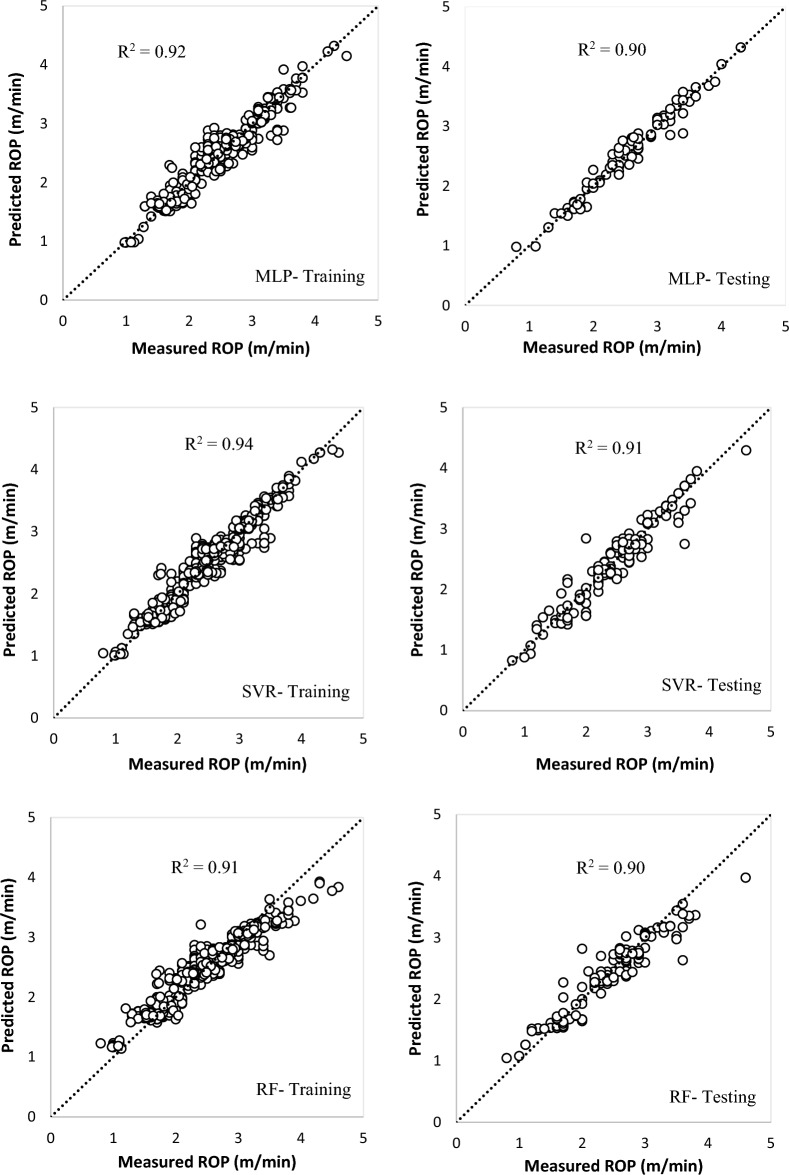
Correlations of predicted ROP versus the measured ROP for machine learning models.

The architectures of machine learning models can be controlled by hyperparameters, but there are no hard and fast rules for selecting the optimal hyperparameters, as the ideal model structure can differ depending on the application. To find the best hyperparameter combinations, researchers often define a grid and employ cross-validation techniques. The current study also uses this methodology. To obtain an optimal network structure with the most suitable hyperparameters, multiple networks were constructed and their results were compared to determine the best one. The overall outcomes of the finest constructed networks are presented in Table 7. The selection and prediction of the ANN model involve several important factors including feature selection, network architecture, and transfer of functions across layers, along with the choice of the training algorithm. Eventually, a network with 7 hidden layers (as depicted in Fig. 7) was chosen. The transfer function used in the hidden layers is hyperbolic tangent, while the output layer employs the exponential transfer function. Cross-correlations graphs of MLP, SVR, and RF models are presented in Fig. 8. The results and performance indices of developed models are presented in Table 8.

**Table 7:** Hyperparameter grid search for Multilayer perceptron, support vector machines and random forests ROP models.

Multilayer perceptron Grid		Best hyperparameter
Solver	SGD, Adam, LBFGS	LBFGS
Number of Neurons in the Hidden layer	3, 4, 5, 6, 7, 8, 9, 10, 11	7
Activation function	Logistic, Tanh, exponential	tanh
l2 Regularization (α)	0.0001, 0.001, 0.01, 0.1	0.001

Support vector machines grid		Best Hyperparameter
Kernel function	Linear, 3rd Degree Polynomial, Gaussian	Gaussian
Epsilon (ε)	0.01, 0.1, 1, 10	0.1
Budget (C)	1, 10, 100	10
Kernel coefficient (γ)	0.1, 0.2, 0.5	0.2

Random Forests Grid		Best Hyperparameter
Number of Trees	50, 100, 150, 300, 500	150
Number of. Features	3, 4, 5	5

**Table 8:** The results and performance indices of developed models.

Model type	Training set					Testing set				
	R^2^	RMSE	MAE	MAPE	VAF	R^2^	RMSE	MAE	MAPE	VAF
NLMR	0.87	0.25	0.19	8.81	85.17	0.86	0.25	0.19	9.8	83.45
MLP	0.92	0.16	0.11	5.34	92.36	0.90	0.22	0.14	6.94	91.29
SVR	0.94	0.15	0.11	4.84	94.13	0.91	0.19	0.13	6.02	91.11
RF	0.91	0.20	0.15	6.60	90.52	0.90	0.22	0.16	7.20	89.49

## Sensitivity analysis

To recognize the most sensitive factors affecting penetration rate, the cosine amplitude method (CAM) was utilized. To apply this method, all the data pairs are expressed in a common X-space which is used to construct a data array X, defined as^74^:

8 $${\text{X}}=\{{{\text{X}}}_{1}, {{\text{X}}}_{2}, {{\text{X}}}_{3} ,\dots . {{\text{X}}}_{{\text{n}}}\}$$

Each of the elements, Xi, in the data array X is a vector of lengths of n, that is:

9 $${X}_{i}=\{{{\text{X}}}_{{\text{i}}1}, {{\text{X}}}_{{\text{i}}2}, {{\text{X}}}_{{\text{i}}3} ,\dots {{\text{X}}}_{{\text{in}}}\}$$

Thus, each of the data pairs can be thought of as a point in n dimensional space, in which each point requires n coordinates for a full description. Each element of the relation, rij, results in a pairwise comparison of two data pairs. The strength of the relation between the data pairs, x_i_ and x_j_, is given by the membership value expressing the strength:

10 $${{\text{R}}}_{{\text{ij}}}= \frac{{\sum }_{{\text{k}}=1}^{{\text{n}}}({{\text{x}}}_{{\text{ik}}} \times {{\text{x}}}_{{\text{jk}}})}{\sqrt{{\sum }_{{\text{k}}=1}^{{\text{n}}}{{\text{x}}}_{{\text{ik}}}^{2} \times {\sum }_{{\text{k}}=1}^{{\text{n}}}{{\text{x}}}_{{\text{jk}}}^{2}}}$$

where i, j, and k represent respectively the counters of the number of input indicators in each data series, the indicators or factors related to each data series, and the number of data series or samples. The closer Rij is to one, the greater the impact of the input indicator on the target index. If there is no effect, the value of Rij will be zero.

**Figure 9:**
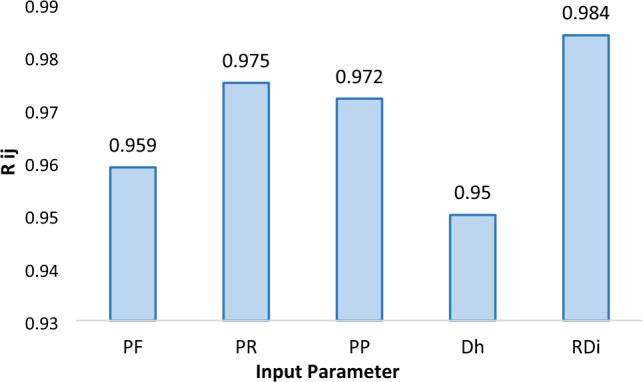
Strengths of relation (R_ij_) between the penetration rate and each input parameter.

The strengths of relations (rij values) between the ROP and input parameters are shown in Fig. 9. Considering that the values of Rij are high for all parameters affecting the penetration rate, and based on the graph presented in Fig. 9, it can be concluded that all of the considered parameters are significantly involved in the rate of penetration. As it is shown, the most effective parameter on the ROP is Rock mass drillability index. In other words, the characteristics of the rock mass have a great influence on the drilling speed.

## Results and discussion

The application of an index that reflects the general characteristics of the rock mass can be instrumental in expediting the estimation of rock mass characteristics and reducing costs. The rock mass drillability index (RDi) possesses this ability and can prove beneficial in penetration rate studies. However, before utilizing any index or parameter in the field of modeling for prediction, it is imperative to establish the effect of that parameter on the output result. Since the RDi has not been implemented in underground mines and underground drilling techniques differ from open pit mining, it is crucial to first demonstrate the efficacy of the RDi in underground drilling and investigate its effect on the penetration rate. After studying the relationship between the penetration rate and RDi, the index was considered as one of the inputs of the model that represents the characteristics of the rock mass.

The dataset utilized for calculations comprises 737 rows. Initially, missing values are eliminated. Subsequently, the IQR method is applied to detect outliers and bad data readings, and they are replaced with the parameter's average value to neutralize their effects. The resultant dataset comprises 737 rows, which are normalized using the min–max normalization method. Thereafter, the dataset is randomly split into 80–20% train and test subsets. The machine learning hyperparameters are fine-tuned utilizing the grid search range srategies, and the optimal hyperparameters are listed in Table 7.

Various metrics can be employed to evaluate the accuracy of the desired model in approximation. For instance, smaller values of MAE, MAPE, and RMSE indicate a higher accuracy in approximation, whereas bigger values of R^2^ and VAF indicate the same. Furthermore, the MAPE values can be used to calculate the absolute value of the average percent relative error, which provides a comprehensive analysis of the model's performance. In addition, R^2^ is instrumental in determining the percentage of the model outputs that can be defined by the fitted line on the data points, and a value of R^2^ close to one is indicative of good accuracy in approximation.

The development of regression models and subsequent comparison between them revealed that the relationship between the penetration rate and its predictive parameters is nonlinear. Due to the potential for complex nonlinear relationships that regression models cannot extract between data, methods based on artificial intelligence were employed to develop the model. Upon analysis of the training and test data results, it was discovered that machine learning models exhibit higher accuracy and lesser error than regression models. This highlights the veracity of the assumption regarding the existence of complex relationships between the parameters that predict penetration rate. Thus, it can be inferred that mathematical models are comparatively less accurate than computational intelligence-based models. The mathematical models assume that the effect of some drilling variables on ROP has a linear and absolute incremental behavior. Upon scrutinizing the models generated through the machine learning method, it is evident that the SVR method outperforms the other models in terms of higher accuracy and lesser error.

To compare the results obtained from the machine learning (ML) models with the previous Rate of Penetration (ROP) models, a ROP model (Shen model) was selected, the mathematical relations of which are depicted in Eq. (11)^68^.

11 $$ \upsilon = - 0.{\text{77p}}_{{\text{t}}} \left( {0.{\text{5p}}_{{\text{e}}}^{2} - {15}.{\text{7 p}}_{{\text{e}}} + {117}.{6}} \right) $$

where ʋ denotes the rate of penetration (mm/s), ${{\text{p}}}_{{\text{t}}}$ is Propelling pressure (MPa) and ${{\text{p}}}_{{\text{e}}}$ is Percussive pressure (MPa).

**Figure 10:**
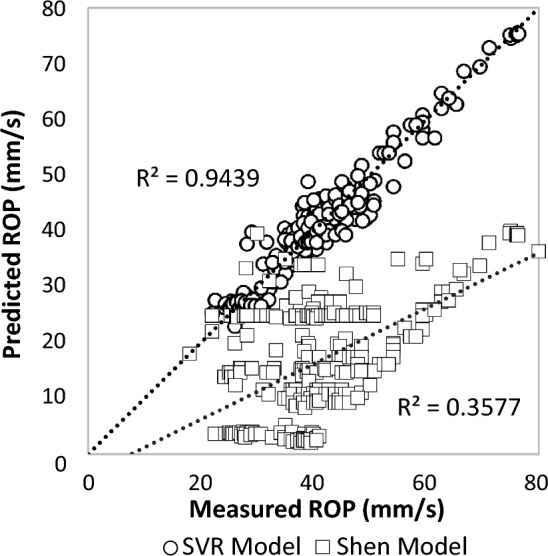
Correlations of predicted ROP versus the measured ROP for Shen model vs. SVR model using data set.

A comparison was then conducted between these models using the database. Further details regarding these models can be found in the relevant research literature^68^. Figure 10 illustrates the outcomes of the previous model, demonstrating the prediction error. It is evident from the figure that this model poorly fits the data, exhibiting high error and significant deviation in ROP values. This inadequacy stems from the model's failure to account for the effect of rock mass discontinuities. Also, the model developed in this research provides better results due to the use of more parameters. Based on the observations from Fig. 10, it can be inferred that the mathematical models are notably less accurate when contrasted with the computational intelligence-based models.

## Conclusions

Efficient drilling processes can lead to cost savings through increased penetration rates. Optimizing drilling processes requires a thorough understanding of the interplay between various parameters affecting the process. Estimating the penetration rate factor enables engineers to better plan for the future and adjust drilling parameters for optimal results. To prepare a suitable database, effective parameters affecting the Rate of Penetration (ROP), including rock mass properties and machine specifications for 737 boreholes in the mentioned underground mines, were collected and datasets were divided randomly into training (80%) and testing (20%) datasets. These parameters served as model inputs to predict ROP. By utilizing machine parameters and rock properties, multiple regression analyses and machine learning methods were implemented, revealing their dominant effect on jumbo drill performance. These algorithms attempt to learn the physical behavior between independent and dependent parameters based on the underlying theory. The learned relation can then be generalized to predict system behavior. In summary, the following outcomes can be drawn from this research:

- Several simple regression analyses were conducted, determining correlations between different variables and penetration rate. Results showed that determination coefficients of simple regression analyses were rather low.
- Regression models were developed with high correlation coefficients. The regression model for jumbo drill penetration rate (Eq. 7) can effectively predict rock drill performance by utilizing rock properties and machine parameters. Other proposed models (Table 6) for predicting penetration rate were also found to be reliable.
- Machine learning algorithms demonstrated better results than regression algorithms for predicting ROP.
- The Support Vector Regression (SVR) model exhibited good predictive results for both training and testing databases compared to other machine learning models. SVR also had the lowest error rate compared to Random Forest (RF) and Multi-Layer Perceptron (MLP) methods. However, the use of these methods may be conditional.
- According to the sensitivity analysis of the effective parameters in the penetration rate, it can be concluded that all of the considered parameters significantly contribute to the rate of penetration.The most influential parameter on the ROP is the Rock Mass characteristic.

The Rock Drillability Index (RDi) being unaffected by rock type suggests that the prediction models developed in this study can estimate jumbo drill performance across different challenging rock conditions. While our model shows promise for application in dry and semi-dry rock formations, it's essential to recognize its limitations in saturated rock conditions and high-water-volume mines, as these scenarios may introduce complexities not accounted for in the model, potentially affecting prediction accuracy. Additionally, the model's applicability may be limited in less jointed rock masses, particularly those lacking cohesion, such as homogeneous rock formations, posing challenges for accurately predicting drilling rates. Furthermore, our model is tailored specifically for underground drilling conditions, and its effectiveness in scenarios involving drilling in open pits or larger diameter holes may be compromised due to distinct challenges not addressed in its design. The prediction models established in this research can be further refined, suggesting significant possibilities for drilling automation in underground mines. Hence, the findings of this study hold great potential for the field of drilling automation in underground mines.

## Data Availability

The datasets used and analyzed during the current study available from the corresponding author on reasonable request.
